# A fat mass and obesity-associated gene polymorphism influences fat mass in exercise-trained individuals

**DOI:** 10.1186/s12970-018-0246-7

**Published:** 2018-08-08

**Authors:** Jose Antonio, Sarah Knafo, Ritishka Kapoor, Jaime L. Tartar

**Affiliations:** 1 Department of Health and Human Performance, Nova Southeastern University, 3401 South University Drive, Davie, FL 33328 USA; 2 Department of Psychology and Neuroscience, Nova Southeastern University, Davie, FL USA

**Keywords:** DXA, FTO, Genotype, Athlete

## Abstract

**Background:**

A single nucleotide polymorphism (SNP) in the fat mass and obesity-associated (FTO) gene is a strong predictor of obesity in humans. The FTO SNP (rs1421085) results in a T to C nucleotide substitution that may result in an increased risk for obesity in individuals who carry at least one C allele. The purpose of this investigation was to characterize the FTO genotype in a cohort of exercise-trained men and women.

**Methods:**

We tested 108 exercise-trained individuals that included professional mixed martial arts fighters, competitive distance runners, collegiate swimmers, stand-up paddlers as well as a cohort of recreational bodybuilders. Body composition was assessed via dual-energy x-ray absorptiometry (DXA). Saliva samples were collected in order to genotype participants and quantify cortisol levels.

**Results:**

The physical characteristics of the subjects were as follows (mean±SD): body weight 74.5±15.6 kg; height 171.5±9.5 cm; bone mineral content 2.8±0.7 kg; fat mass 15.7±5.5 kg; lean body mass 55.9±14.4 kg; % body fat 21.6±7.0. Independent samples t tests showed that C allele carriers (*n* = 54) had significantly higher fat mass t(106) = 3.13, *p* < 0.01 and body fat percentage t(106) = 2.68, p < 0.01, relative to the TT group (n = 54) (i.e., fat mass: C/− 17.3 ±5.6 kg, TT 14.2±4.6 kg; body fat percentage: C/− group 23.4±7.4%, TT group 19.9±6.2). No other measures of body composition were associated with the FTO genotype (i.e., body mineral density, bone mineral content, or lean body mass). Moreover, cortisol levels were significantly higher in the TT group relative to the C allele carriers t(106) = 2.37, *p* = 0.02 (i.e., TT 0.35 ±0.35 μg/dL, C/− 0.22±0.16 μg/dL).

**Conclusions:**

Our findings demonstrate a relationship between C allele carriers on the FTO gene and a predisposition to a higher fat mass and body fat percentage. In addition, we found no relationship between cortisol and fat mass. However, due to the cross-sectional nature of this investigation, we cannot infer causality regarding the FTO gene and body composition.

## Background

There is a powerful genetic influence on fat mass with heritability estimates ranging from 40 to 80% [1]. In particular, polymorphisms in the fat mass and obesity associated (FTO) gene are related to individual differences in food intake and energy balance [2] and can also influence skeletal muscle phenotype [3]. Multiple single nucleotide polymorphisms (SNPs) occur on the FTO gene that may influence adipogenesis and obesity [4–6]. Since the obesity-associated SNPs are on the intron 1 region of the FTO gene, the mechanisms through which they influence body mass are uncertain. However, it has recently been shown in humans that a T-C SNP at position 53,767,042 on the FTO gene (rs1421085) causes an increase in IRX3 and IRX5 protein expression during early adipocyte differentiation in favor of energy-storing/white adipocytes over energy-dissipating/beige adipocytes. The critical downstream effect of this is increased energy conservation in the form of augmented fat storage [7].

Previous work has shown that exercise intervention can ameliorate the influence of FTO risky alleles on BMI on the FTO rs8050136 SNP [8, 9] and the rs9939609 SNP [10–12]. However, due to the direct influence of the FTO SNP rs1421085 on early adipocyte differentiation, the effect of exercise training on body fat in the risky allele carriers of the rs1421085 SNP are less certain. The purpose of the study was to examine the relationship between FTO genotype and body composition in trained individuals. We also determined if salivary cortisol is related to fat mass in exercise-trained individuals.

## Methods

### Participants

Study subjects came to the laboratory on a single occasion for body composition assessment and the provision of a saliva sample. In accordance with the Helsinki Declaration, the university’s Institutional Review Board approved all procedures the involved human subjects. Written informed consent was obtained prior to participation. All testing took place between 1130 and 1400. Participants were instructed not to exercise or eat or drink anything other than water one hour prior to testing.

### Body composition

One hundred and eight subjects had their height and weight determined using a calibrated scale. Body composition was assessed via dual-energy X-ray absorptiometry (DXA) (Model: Hologic Horizon W; Hologic Inc., Danbury CT USA). Quality control calibration procedures were performed on a spine phantom. Subjects wore typical athletic clothing and removed all metal jewelry. They were positioned supine on the DXA within the borders delineated by the scanning table. Each whole body scan took approximately seven minutes.

### Genotyping

Genomic DNA was extracted in a QIAcube instrument following the manufacturer’s standard protocol for saliva nucleic acid extraction (QIAGEN, Valencia, CA). After isolation, allelic discrimination for the FTO gene was determined via real-time polymerase chain reaction (PCR) using a TaqMan SNP genotyping assay using fluorogenic probes (Applied Biosystems, CA) with the primer sequence TAGCAGTTCAGGTCCTAAGGCATGA[C/T]ATTGATTAAGTGTCTGATGAGAATT. Thermal cycling was performed on StepOne Real-Time PCR system (Applied Biosystems, CA). The amplification mix contained the following ingredients: 12.5 μL of PCR master mix (QIAGEN, Valencia, CA), 1.25 μL of TaqMan 20X working stock, 10.25 μL of RNase- and DNase-free water (Sigma), and 1.0 μL of sample DNA, in a total volume of 25 μL per single tube reaction. The PCR conditions were 95 °C for 10 min followed by 40 repeated cycles of 95 °C for 15 s and 60 °C for 60 s. Aliquots corresponding to the products from the first round of amplification were used as templates for a second round of amplification (30 cycles, with the same conditions). Genotypes were determined automatically via the StepOne software (Applied Biosystems, CA) based on the fluorescence signals. Samples were run in duplicate and in the case of a call discrepancy, samples were rerun.

### Cortisol

Saliva samples were run in duplicate and quantified via a human cortisol enzyme immunoassay (EIA) kit per the manufacturer’s instructions (Salimetrics LLC, USA). The samples were immediately read in a BioTek ELx800 plate reader (BioTek Instruments, Inc., USA) at 450 nm with a correction at 630 nm. All samples were within the detection ranges indicated in the immunoassay kits. The variation of sample readings was within the expected limits and the average intra-assay coefficient of variation was 4.76%. Final concentrations for the biomarkers were generated by interpolation from the standard curve in μg/dL.

### Statistical analysis

We conducted a series of independent samples t-tests to assess the relationship between FTO genotype and DXA body composition measures. An independent samples t-test test was also run to determine a possible relationship between FTO genotype and cortisol levels. Significant findings were followed up with 2 × 2 ANOVAs in order to test for possible sex x genotype interactions. The distribution of allele frequencies was determined by the Hardy–Weinberg Exact (HWE) test; moreover, the association of allele status was analyzed using the chi-square test. All calculations were conducted using an SPSS statistical package (version 19, SPSS Inc., IBM). All reported *p*-values are two-tailed with a priori significance level of *p* < 0.05.

## Results

The physical characteristics of the subjects were as follows (mean±SD): body weight 74.5±15.6 kg; bone mineral content 2.8±0.7 kg; fat mass 15.7±5.5 kg; lean body mass 55.9±14.4 kg; body fat percentage 21.6±7.0%. The population that we studied consisted of the following: 41 resistance-trained individuals (primarily recreational bodybuilders), 22 competitive stand-up paddlers/rowers, 15 professional fighters (i.e., MMA, boxing), 11 collegiate division II swimmers, 9 collegiate division II track and field athletes, 8 collegiate division II distance runners, 1 division II collegiate soccer player and 1 distance cyclist. Thus, 42 and 66 subjects primarily performed aerobic and anaerobic exercise, respectively. The percentage of the TT and C/− carriers that were aerobic athletes was 40% and 42%, respectively. Thus, no differences existed regarding the distribution of athlete type (aerobic versus anaerobic).

### Genotype frequency

FTO genotype frequencies were as follows: 14% CC, 37% AG, and 49% GG. The HWE test showed that χ2 = 2.73, *p* > 0.05, suggesting that the population is consistent with Hardy-Weinberg Equilibrium, and confirming that the allele types were randomly sampled. Because previous research suggests carrying one C allele in rs1421085 associates with an obesity risk [13, 14], we collapsed across genotypes containing the C allele. The CC homozygotes and the CT heterozygotes (*n* = 54, 25 males) were compared to the TT homozygotes (n = 54, 31 males).

### Association between FTO and body composition measures

**Fig. 1:**
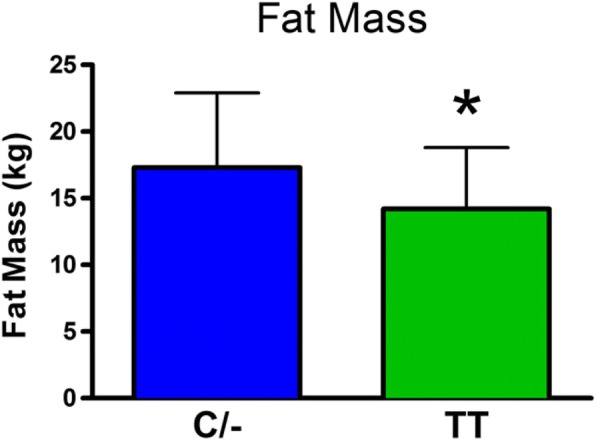
The relationship between fat mass and genotype. Fat mass was significantly lower in the TT group (*p* = 0.002). (Mean±SD)

**Fig. 2:**
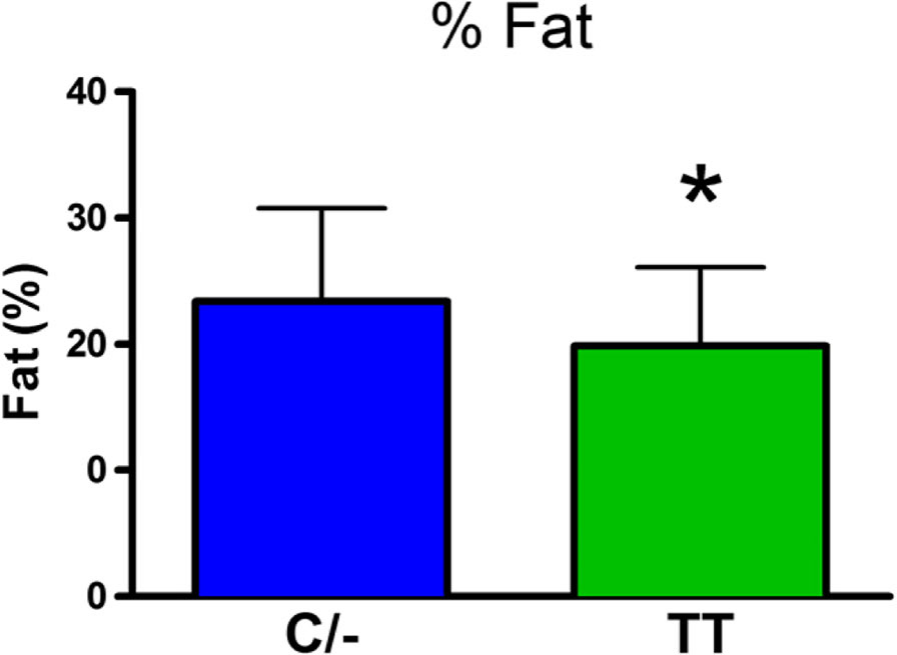
The relationship between percent body fat and genotype. Body fat % was significantly lower in the TT group (*p* < 0.01). (Mean±SD)

**Fig. 3:**
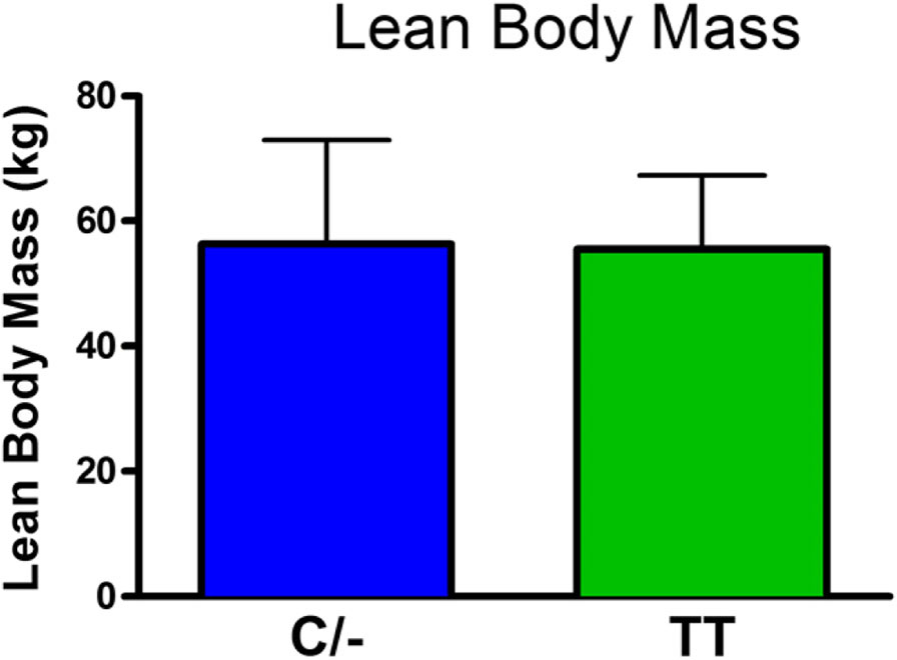
The relationship between lean body mass and genotype. There were no significant differences in lean body mass. (Mean±SD)

**Fig. 4:**
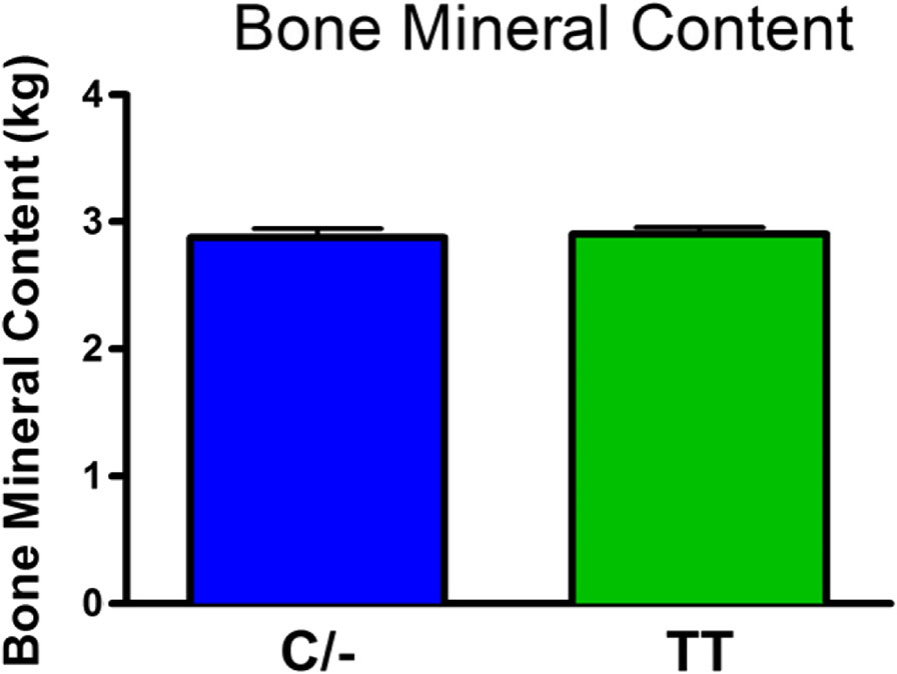
The relationship between bone mineral content and genotype. There were no significant differences in bone mineral content. (Mean±SD)

**Fig. 5:**
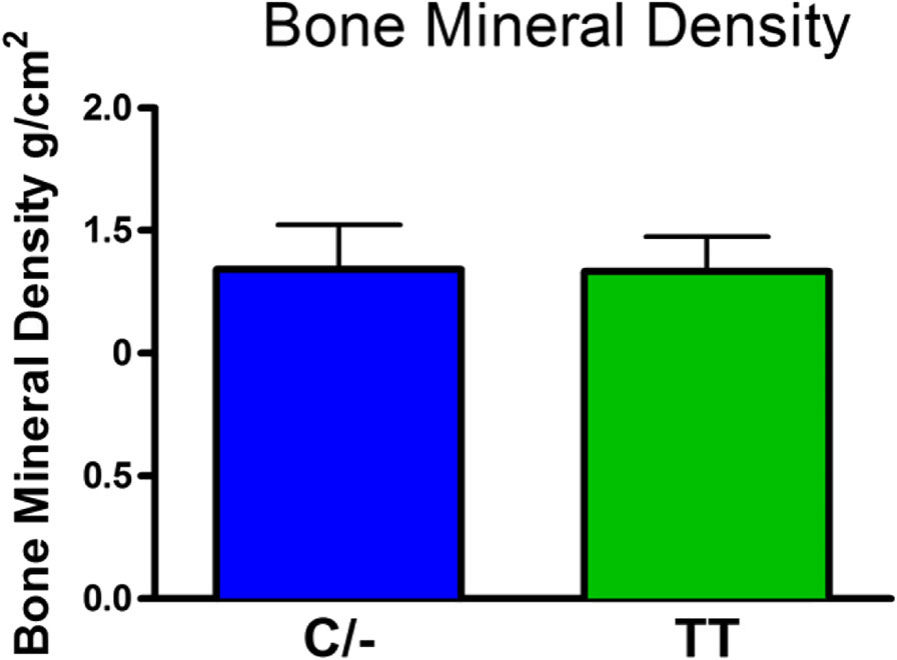
The relationship between bone mineral density and genotype. There were no significant differences in bone mineral density. (Mean±SD)

**Fig. 6:**
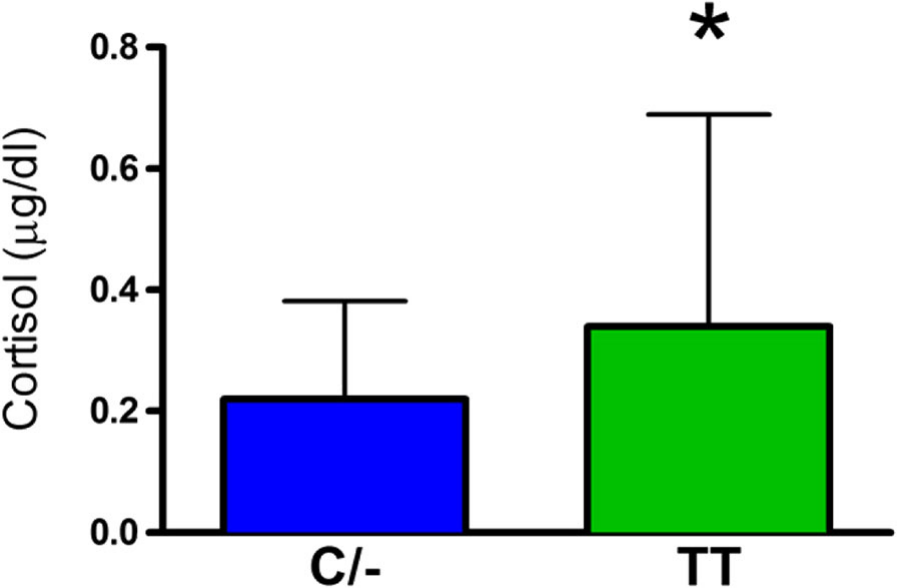
The relationship between salivary cortisol and genotype. The TT group had significantly higher cortisol (*p* = 0.02). (Mean±SD)

There was no significant difference in body weight between the C/− and TT groups (mean±SD: C/− 75.3±17.1 kg and TT 70.7±12.2 kg). The role of the FTO genotype on body composition is provided in Figs. 1, 2, 3, 4 and 5. The FTO allele associated with obesity was significantly higher on two measures of body fat. Specifically, fat mass was significantly greater t(106) = 3.13, *p* = 0.002 in the C/− group (17.3 ±5.6 kg) relative to the TT group (14.2±4.6 kg) (Fig. 1). Body fat percentage was significantly greater t(106) = 2.68, *p* = 0.009 in the C/− group (23.4±7.4%) relative to the TT group (19.9±6.2 5) (Fig. 2). There were no between group differences for lean body mass, bone mineral content, and bone mineral density (Figs. 3, 4 and 5). We also ran a follow up 2 X 2 analysis of variance to probe for sex by genotype effects. This test showed no significant sex X genotype interaction for body fat percentage F(1, 104) = 0, *p* = 0.99) or total fat mass F(1, 104 = 3.18, *p* = 0.08). Results for cortisol revealed that the TT group had significantly higher t(106) = 2.36, *p* = 0.02 cortisol (0.35 ±0.35 μg/dL) relative to the C/− group (0.22±0.16 μg/dL) (Fig. 6).

## Discussion

The current investigation found that C allele carriers had significantly higher fat mass and a body fat percentage relative to the TT group. Thus, even in a group of highly trained individuals, the risk allele was associated with higher body fat. One could speculate that in spite of intense exercise training, C allele carriers may still have greater fat mass than TT individuals. One might suggest that those who perform primarily endurance activities tend to carry less fat (i.e., fat mass and percent body fat). However, that was not the case in our investigation. We had an equal percentage of athletes that performed aerobic or anaerobic activities in the C/− and TT groups.

There is a dearth of studies on the FTO gene as it relates to an athletic or exercise-trained population. Eynon et al. found that the FTO A/T polymorphism was not associated with elite athletic status in a large cohort of European athletes (i.e., 266 endurance and 285 sprint/power athletes) [15]. Certainly this makes sense inasmuch as athletic performance, particularly in skill sports, cannot be predicted solely on the basis of body composition. Huuskonen et al. studied 846 healthy Finnish males and found that aerobic fitness does not modify the effect of FTO rs1421085 variation on body composition traits [16]. This is in agreement with our data showing that the percentage of endurance-trained individuals was equally represented in the TT and C/− genotypes. Heffernan et al. genotyped a group of non-athletes and elite rugby players for the FTO gene [17]. They discovered that T allele carriers had greater total body and total appendicular lean mass versus the AA genotype. Furthermore, the T allele was more common (94%) in selected elite rugby union athletes. Thus, at least in comparing athletes to non-athletes, the TT genotype is ostensibly more common.

A further aim of the study was to show whether cortisol is a pathway through which C allele carriers have greater levels of body fat. This possibility has arisen, at least in part, because previous work has shown that exercise increases cortisol levels [18] and there exists an association between high cortisol levels and obesity [19]. Interestingly, we found that the TT FTO rs1421085 group had significantly higher cortisol levels than the risky C/− group. Given that the TT group had significantly lower body fat percentage and fat mass than the C/−group, this finding supports that notion that cortisol is not related to increased fat mass in exercise-trained individuals. Although it is unclear why the TT genotype relates to higher cortisol levels, one might speculate that the TT group has a greater ratio of brown to white adipose tissue than the C/− group. This could result in higher cortisol levels due to the increased energy need in the form of increased calorie expenditure [7]. In support of this idea, a previous study found that higher cortisol levels were associated with increased activity in brown adipose tissue [20]. However, while this idea is intriguing, more work will need to be done to clarify the relationship between FTO rs1421085 genotype, brown vs. white adipose tissue, and cortisol.

A major limitation of our investigation is the cross-sectional design. For instance, it would be intriguing to see if exercise-trained individuals that are TT can lose more fat mass than their C/− counterparts when given the same hypocaloric diet. Based on the cross-sectional data from our investigation, one would predict that TT individuals should lose more fat mass, similar to a previous study that investigated a different FTO SNP [21] since the common FTO polymorphisms all associate with obesity and there is high linkage disequilibrium between them [22, 23]. In particular, Zhang et al. tested the effect of FTO variant on weight loss in response to 2-year diet interventions in 742 obese adults [21]. They discovered that carriers of the risk allele of the FTO variant rs1558902 had a greater reduction in weight, body composition, and fat distribution in response to a high-protein diet. Thus, at least in this cohort of obese individuals, a high protein diet may indeed be optimal choice as it relates to changes in body composition. Whether such a strategy would work for an exercise-trained population that has a low body fat percentage is unknown. Future work is needed to elucidate the role of protein intake on modifying body composition in exercise-trained individuals.

## Conclusion

In summary, the cross-sectional data from this investigation suggest that in spite of intense athletic training, C allele carriers (FTO gene, rs1421085) tend to have a greater percentage of fat mass; however, it should be noted that the C/− group in our investigation still have quite low body fat percentages. Thus, it is clear that exercise training can dramatically modify one’s phenotype despite a purported genetic limitation.

## Abbreviations

DXA: Dual energy x-ray absorptiometry
EIA: Enzyme immunoassay
FTO gene: Fat mass and obesity associated gene
Kg: Kilogram
MMA: Mixed martial arts
Nucleotides A, T, C, G stand for: Adenine, thymine, cytosine, guanine
SNP: single nucleotide polymorphism
